# Patients Who Use Multiple EDs: Quantifying the Degree of Overlap between ED Populations

**DOI:** 10.5811/westjem.2015.1.22838

**Published:** 2015-03-17

**Authors:** Baruch S. Fertel, Kimberly W. Hart, Christopher J. Lindsell, Richard J. Ryan, Michael S. Lyons

**Affiliations:** * Emergency Services Institute, Cleveland Clinic Foundation, Cleveland, Ohio; † University of Cincinnati, Department of Emergency Medicine, Cincinnati, Ohio; ‡ University of Cincinnati, Center for Clinical and Translational Science and Training, Cincinnati, Ohio

## Abstract

**Introduction:**

The degree to which individual patients use multiple emergency departments (EDs) is not well-characterized. We determined the degree of overlap in ED population between three geographically proximate hospitals.

**Methods:**

This retrospective cohort study reviewed administrative hospital records from 2003 to 2007 for patients registered to receive ED services at an urban academic, urban community, and suburban community ED located within 10 miles of one another. We determined the proportion who sought care at multiple EDs and secondarily characterized patterns of repeat encounters.

**Results:**

There were 795,176 encounters involving 282,903 patients. There were 89,776 (31%) patients with multiple encounters to a single ED and 39,920 (14%) patients who sought care from multiple EDs. The 39,920 patients who sought care from multiple EDs generated 185,629 (23%) of all encounters. Patients with repeat encounters involving multiple EDs were more likely to be frequent or highly frequent users (30%) than patients with multiple encounters to a single ED (14%).

**Conclusion:**

While only 14% of patients received care from more than one ED, they were responsible for a quarter of ED encounters. Patients who use multiple EDs are more often frequent or highly frequent users than are repeat ED visitors to the same ED. Overlap between ED populations is sufficient to warrant consideration by multiple domains of research, practice, and policy.

## INTRODUCTION

Patients often seek emergency services on more than one occasion, and patterns of repeat utilization within a single emergency department (ED) are increasingly reported.^1^–^5^ The degree to which patients may visit multiple EDs, as opposed to using a single ED multiple times, is less characterized. Several studies have shown that some patients use multiple EDs within a relatively short time period,^6^–^8^ but most attention has been focused on ED patients that use the ED frequently.^9^,^10^ The magnitude of multiple ED use over longer periods (i.e. >1 year) has not been explored, and only one study^11^ has reported the frequency of multiple ED use by ED patients who are not frequent users. Similarly unknown is whether persons who use multiple EDs differ from those who frequent the same ED multiple times.

Overlap in patient populations between multiple EDs could have broad implications for regional planning of ED service capacity, interventions targeting repeat ED utilization, ED market share calculations that use patients rather than encounters as the unit of analysis, community-wide follow-up in research studies, public health intervention, and health information systems.^1^,^12^–^18^ In this exploratory report using data from three hospitals, we estimate the proportion of ED patients who seek care using a sample of three geographically contiguous mostly adult EDs in a region with a total of 18 EDs and one dedicated pediatric ED and describe patterns of multiple ED use over a five-year period. Secondarily, we explore differences in the population that visits multiple EDs versus the population that uses only one ED and also the degree to which frequent and highly frequent ED users contribute to multiple ED utilization.

## METHODS

This multi-center, retrospective cohort study involved automated electronic query of hospital administrative databases. We included all patients who were registered to receive ED services at any of the three study site hospitals. The study was institutional review board approved.

We obtained data from an urban academic, urban community, and suburban community hospital, all located within 10 miles of each other. The urban facilities were less than two miles from each other. These facilities cared almost exclusively for adult patients, as a large pediatric hospital is nearby. Each ED hosted research and residency training and was staffed by the same emergency physician group. In 2007, at the end of this study period, the surrounding county had a population of 855,062 that was 72% white, 25% black, 2% Asian, and 2% Hispanic. These demographics were stable throughout the study period.

During the study period, the hospitals were partnered in terms of purchasing, information technology, and other operational support, but they were owned separately and generally perceived to have distinct patient populations and differing missions. The hospitals were open to all patients, with no regional payer exclusions and no structured referral system. Fifteen other EDs in the metropolitan area were not included in the study.

Hospital administrators directly exported an electronic data set of ED encounters from billing databases using a standardized query. Data were available for all three hospitals from 2003 to 2007. Individuals presenting for care were registered using date of birth, social security numbers, names, and government identification. Patients were issued a unique medical record number at their first encounter that remained static across time and across facility. Each separate encounter generated a unique account number, which was linked to information about when and where that encounter occurred. To facilitate billing and reconciliation, the hospitals conducted ongoing internal quality assurance review that included merging records, purging of duplicate records, and updating of names.

Upon receiving the exported data set, we considered each unique medical record number to represent a unique patient. Each patient was then considered to have had one or more ED encounters, determined by the number of unique account numbers linked to each unique medical record number. We then categorized patients with multiple encounters as to whether their encounters involved only one ED, or occurred at multiple EDs.

The primary outcome was the proportion of all ED patients across study hospitals who sought care at multiple EDs. Secondary outcomes included demographics for single and multiple ED users, distribution of encounters between sites, duration of time between encounters at different EDs by the same patient, the proportion of ED encounters that were from patients who were multiple ED users, and the proportion of frequent and highly frequent ED users who sought care at more than one ED. To classify patients based on frequency of ED use, we used the commonly considered metric of number of encounters within a calendar year. Specifically, we assigned patients as being single users (1 encounter), repeat users (2–3 encounters), frequent users (4–7 encounters), or highly frequent users (8 or more encounters) based on the year in which they had the most encounters. We performed data management and analyses using SPSS 21.0 (IBM Corporation, Armonk, NY).

## RESULTS

There were 795,176 encounters by 282,903 patients for the three EDs from 2003 to 2007. The median number of encounters per patient was two (range 1–352, IQR 5). There were 443,593 (56%) encounters at the academic ED, 184,874 (23%) at the urban community ED, and 166,709 (21%) at the suburban ED.

### Proportion of Patients Seeking Care at Multiple EDs

Of the 282,903 patients in the study, 39,920 (14%) sought care at more than one facility during the five-year period. Figure depicts the proportion of patients seen by each ED or various combinations of EDs. Table 1 shows patient-level demographics at the first visit to the system for the patient cohort overall, the subset of patients with ED encounters involving multiple facilities, and the subset with either single or multiple encounters involving only one ED.

### Patterns of ED Use

There were 153,207 (54%) patients with only a single encounter and 129,696 (46%) patients with multiple ED encounters during the study period. Of those with multiple ED encounters, 89,776 (69%) patients used only one ED and 39,920 (31%) used more than one ED.

Repeat ED users who visited only one ED accounted for 456,340 (57%) of all encounters. Patients had a median of three encounters to that ED (range 2–344, IQR 3) over the five-year study period. Patients with repeat ED encounters involving more than one ED accounted for 185,629 (23%) of all encounters. These patients had a median of six encounters across all EDs (range 2–352, IQR 9). The median number of days between the first encounter and second encounter at the ED was 393 days (0 to 1,824 days, IQR 656).

### Multiple ED Use by Frequent Users of ED Services

Table 2 shows patterns of single and multiple ED use stratified by frequency of ED use. Of the 282,903 patients in the study, 20,144 (7%) were frequent users, with at least four visits in any single year, and 4,901 (2%) were highly frequent users, with at least eight visits in any single year. Of frequent users, 9,119 (45%) presented to multiple EDs. Of highly frequent users, 3,098 (63%) presented to multiple EDs. Frequent and highly frequent users who sought care in multiple EDs contributed 57,393 (7%) and 69,514 (9%) encounters respectively. Patients with encounters at multiple EDs were more likely to be frequent or highly frequent ED users compared to patients with multiple encounters to only a single ED (30.6% vs. 14.3%, difference in proportions 16.3%, CI_95_ 15.8.% – 16.8, p<0.0001).

## DISCUSSION

In this longitudinal exploration of the overlap in ED patient populations between hospitals, we found that although patients often visit the ED repeatedly, most repeat encounters occur within a single ED rather than among multiple EDs. Nonetheless, nearly one of every four ED encounters were by patients who also visited another ED during the study period. Overall, our findings indicate there is the potential for both repeat and highly frequent ED use to go unrecognized when looking at only a single hospital. Our results demonstrate the need for large-scale and detailed analyses, inclusive of all EDs in a given region, to fully demonstrate and describe this phenomenon.

Many interesting potential implications of repeat ED use have been discussed previously,^1^,^5^,^11^,^12^ but this discussion has largely focused on repeated use of a single ED. There are many issues likely to result from failure to consider overlapping patterns of ED use and thus underestimating ED use. The costs of ED utilization for any given patient would be greater when considering multiple EDs as would the aggregate costs attributable to repeat rather than single ED use especially in the realm of radiologic and laboratory testing. From a health systems perspective, planning of ED services depends not only on the number of ED encounters but also the number of patients served; this study demonstrates that the number of patients cared for by EDs within a region is less than would be expected if simply adding the number of patients cared for by each ED. Similarly, adding data from multiple EDs will somewhat overestimate the prevalence of disease within a population. Any market-share calculations that consider the number of people using the ED rather than the number of ED encounters would be complicated by the sizeable minority of patients that are shared between EDs. Moreover, research commonly considers return visits to the ED in outcome assessments, which may be underestimated unless multiple EDs are included.

There are also several direct practice implications arising from the phenomena of multiple ED use. Most notably, there is the urgent need for shared information systems with real-time access to improve care and reduce harmful or expensive duplication of services.^10^ Our data also suggest that interventions to coordinate and streamline healthcare (i.e. Accountable Care Organizations, capitated payments, and readmission penalties) should consider multiple ED utilization. Finally, any burden of prevention intervention^13^ or care-coordination^9^,^14^ interventions would be less if shared between hospitals to affect the overall population of ED users within a community.

## LIMITATIONS

Our findings may not be fully generalizable. Data were from a relatively small collection of hospitals within a single region, all of which were characterized by residency programs and non-rural location. Our analysis also did not involve children. Nonetheless, our findings are strengthened by the inclusion of different types of EDs that are sufficiently close to at least partially attenuate transportation barriers. We do not know what other local EDs these patients might have visited, or their motivations to seek care at more than one ED.

Our study does not demonstrate the extent to which using multiple EDs is problematic or inappropriate. We do not know the extent to which encounters were related or if they were at all preventable. In addition, there may be multiple logical explanations for choosing different EDs. For example, insurance coverage may change over time, home or job location can change, or a hospital’s reputation for different patient conditions might sway patient preferences. It may be that use of different EDs depending on circumstances is a strength rather than a weakness of the current healthcare system. The association between more frequent ED users and multiple ED use could arise, at least in part, from the fact that the random chance of using more than one ED would increase as frequency of ED use increases.

The likelihood of overlap for these particular sites may have been biased in either direction by the affiliation of the study-site EDs within a consortium of hospitals. However, we are unaware of any particular insurance patterns or policies that would have influenced a patient’s choice for or against this particular set of hospitals. We note that the affiliation between these hospitals was limited and not well understood by the patient population. For example, most patients would not have known that the same emergency physician group staffed all three hospitals.

Our data are subject to the limitations of existing data sets collected for clinical and administrative purposes, including inaccurate or missing data. Whether some patients had different medical record numbers is likely to be rare given the multiple identifiers collected and maintained by the hospital and the ongoing internal quality checks performed. If some patients did erroneously appear to have different identities at different encounters, this might have been more likely between different hospitals than within single hospitals. Our sample may have included some patients transferred between hospitals. When exploring this possibility, we identified only 332 (0.07%) encounters at the academic facility that occurred within one calendar day of discharge from a community facility with an indication of “discharged to another facility”. This suggests that the number of ED to ED transfers is small relative to the overall number of patients using multiple EDs. Our results may also be skewed by the fact that some patients may have died or moved during the study period, though in gross terms, the region’s population remained steady during the study period.

## CONCLUSION

This manuscript demonstrates that repeat encounters by the same patients are common, and most repeat encounters occur within a single ED rather than among multiple EDs. However, the small amount of patients who visit multiple EDs contribute significantly to overall visits and are more likely to be frequent and highly frequent utilizers of ED care than are those who use only one ED on a repeat basis. The magnitude of population overlap between EDs is sufficient to suggest that research, practice, and policy should move further towards considering emergency departments as a combined system and not as individual units.

## Figures and Tables

**Figure:**
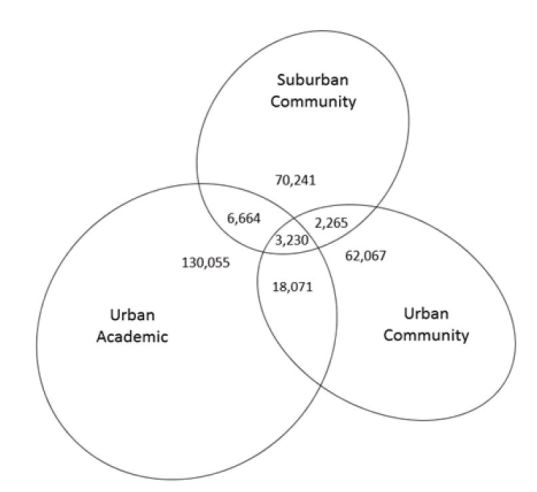
Overlap between emergency department patient populations. Of all patients receiving emergency care (n=282,903), the proportion who were seen in only one emergency department (ED) or in various combinations of multiple EDs over a five-year period. Size of oval and overlapping sections are proportional to number that each section represents.

**Table 1:** Characteristics of emergency department (ED) patients* by utilization of single or multiple EDs.

	Total patients N=282,903		Patients with encounters to multiple EDs N=39,920		Patients with single or multiple encounters to only one ED N=242,983	
	
	N	(%)	N	(%)	N	(%)
Age†	42	(20)	40	(18)	43	(20)
Race						
Caucasian	169,869	(60.0)	18,111	(45.4)	151,758	(62.5)
African-American	91,154	(32.2)	20,653	(51.7)	70,501	(29.0)
Other/not documented	16,126	(5.7)	892	(2.2)	15,234	(6.3)
Hispanic	4,267	(1.5)	172	(0.4)	4,095	(1.7)
Asian	1,487	(0.5)	92	(0.2)	1,395	(0.6)
Sex						
Female	145,846	(51.6)	23,010	(57.6)	122,836	(50.6)
Male	137,050	(48.4)	16,910	(42.4)	120,140	(49.4)
Payor						
Commercial	102,755	(36.3)	10,746	(26.9)	92,009	(37.9)
Self-pay	84,258	(29.8)	13,335	(33.4)	70,923	(29.2)
Medicare	57,449	(20.3)	8,659	(21.7)	48,790	(20.1)
Medicaid	25,467	(9.0)	5,813	(14.6)	19,654	(8.1)
Other	12,974	(4.6)	1,367	(3.4)	11,607	(4.8)

* Includes only first encounter to any ED during the study period; future encounters excluded to avoid patient duplication.

† Presented as mean and (standard deviation).

**Table 2:** Frequency of emergency department (ED) use for patients with multiple ED encounters by use of single or multiple EDs.

	Total patients with multiple ED encounters N=129,696		Patients with encounters to multiple EDs N=39,920		Patients encounters to only one ED N=89,776	
	
	N	(%)	N	(%)	N	%
No more than one visit in any single calendar year	35,309	(27.2)	8,305	(20.8)	27,004	(30.0)
More than one visit in any single calendar year						
Repeat utilizer*	69,342	(53.4)	19,398	(48.6)	49,944	(55.6)
Frequent utilizer†	20,144	(15.5)	9,119	(22.8)	11,025	(12.2)
Highly frequent utilizer‡	4,901	(3.8)	3,098	(7.8)	1,803	(0.2)

*ED,* emergency department

* In at least one year, minimum 2 visits.

† In at least one year, minimum 4 visits.

‡ In at least one year, minimum 8 visits.
